# Malnutrition–Sarcopenia Syndrome and Self-Management Behaviors in Continuing-Care Retirement Community Residents

**DOI:** 10.3390/geriatrics7010009

**Published:** 2021-12-31

**Authors:** Murad H. Taani, Immaculate Apchemengich, Christina Diane Sima

**Affiliations:** 1 College of Nursing, University of Wisconsin Milwaukee, Milwaukee, WI 53211, USA; cdsima@uwm.edu; 2 Joseph J. Zilber School of Public Health, University of Wisconsin Milwaukee, Milwaukee, WI 53205, USA; apcheme2@uwm.edu

**Keywords:** sedentary behavior, aging expectations, protein intake, physical activity

## Abstract

Malnutrition–sarcopenia syndrome (MSS) might put older adults at higher risk for disability, frailty, and mortality. This study examined the prevalence and association of the self-management-process factors (i.e., self-efficacy and aging expectations) and behaviors (protein and caloric intake and sedentary and physical-activity behaviors) to MSS among older adults living in continuing care retirement communities (CCRCs). Using a cross-sectional correlational design, data of 96 CCRC residents (82.4 ± 7.4 years) were analyzed. Muscle mass, strength, function, nutritional status, sedentary time, physical activity levels, protein and caloric intake, self-efficacy for physical activity, aging expectations, and physical and mental health-related quality of life were measured. Results show that 36 (37.5%) had sarcopenia, 21 (21.9%) had malnutrition risk, 13 (13.4%) had malnutrition, and 12 (12.5%) had MSS. We also found that high time spent in sedentary behaviors (OR = 1.041; 95% CI: 1.011–1.071) was associated with higher odds of having MSS and high expectations regarding aging (OR = 0.896; 95% CI: 0.806–0.997) were associated with less likelihood of having MSS. Findings suggest that CCRC residents should be screened for MSS. Self-management interventions that consider the self-management-process factors are needed to prevent MSS and mitigate its negative outcomes among CRCC residents.

## 1. Introduction

Sarcopenia is a syndrome prevalent in older adults and characterized by progressive and generalized loss of muscle strength, mass, and/or function [1]. Sarcopenia is associated with negative health outcomes, including falls, fractures, physical disability, frailty, poor quality of life, and mortality [1,2]. Malnutrition is also a common health problem that affects people from different age groups, particularly older adults [3,4]. Malnutrition is one of the key pathophysiological causes of sarcopenia in older adults [1] and has been linked to many adverse clinical outcomes, including increased hospitalization rates and length of hospital stay, poor muscle quantity and quality, reduced quality of life, and mortality [1,2]. While the prevalence of sarcopenia and malnutrition varies widely depending on the population studied, sex, age, settings, and the diagnostic criteria used, both conditions are highly prevalent among older adults, particularly institutionalized older adults [4,5].

Sarcopenia and malnutrition might occur concurrently among older adults. The clinical presentation of both conditions together has been termed “malnutrition–sarcopenia syndrome (MSS)” [6] and might put older adults at higher risk for disability, frailty, and mortality than those only suffering from either sarcopenia or malnutrition. However, to our knowledge, the concept of MSS has not yet been widely recognized, and research on identifying individuals with MSS, particularly older adults living in continuing care retirement communities (CCRCs) is still lacking in the literature. Further, despite the fact that MSS may lead to numerous and devastating health outcomes for older adults, there is no evidence on the cooccurrence of both conditions and the factors associated with MSS in older adults living in CCRCs.

Older adults living in CCRCs are at higher risk for malnutrition and reduced muscle mass, strength, and function compared to community-dwelling older adults [7,8]. Older adults are typically screened and assessed for either sarcopenia or malnutrition, but rarely for both conditions concurrently [6,9]. As both conditions are amenable to intervention, it is imperative to assess for MSS and its associated factors among this high-risk group who live in CCRCs. Sarcopenia and malnutrition have multifactorial processes where physical activity and nutrient intake exert an important role [6,10]. However, physical activity and dietary self-management behaviors and their antecedents have been overlooked among older adults with MSS living in CCRCs. According to the Individual and Family Self-management Theory (IFSMT) [11], the process of self-management, including knowledge and beliefs, impacts self-management behaviors and both can directly influence health outcomes. Hence, knowledge and beliefs of self-efficacy for physical activity and aging expectations could influence older adults’ self-management behaviors (i.e., engaging in sedentary and physical activity behaviors and eating adequate nutrients including protein and calories) and health outcome (muscle mass, strength, function, and nutritional status). However, these relationships have not been studied in older adults with MSS living in CCRCs. We conducted this study with the following objectives: (1) to identify the prevalence of MSS in older adults living in CCRCs and (2) to examine the relationship of MSS to self-management processes including self-efficacy and aging expectations as well as the sedentary, physical activity, and dietary self-management behaviors in this population.

## 2. Materials and Methods

**Figure 1:**
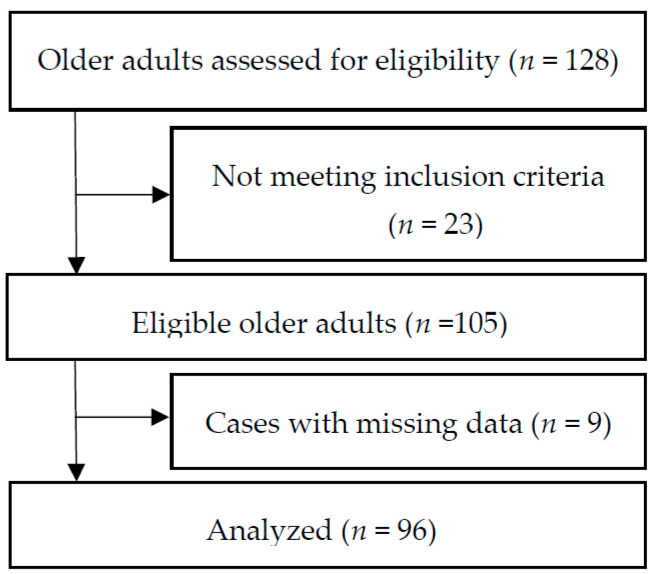
Flowchart of selection for study participants.

We used a cross-sectional correlational design and a convenience sample of 105 older adults living in six CCRCs in the Midwestern United States (Figure 1). Inclusion criteria included: (1) English speaking, (2) >70 years old, and (3) having a score >26 on the Montreal Cognitive Assessment [12] and a score <11 on the 15-item Geriatric Depression Scale [13]. Individuals who were unable to stand or walk without assistance (using assisted de-vices such as canes or walkers was allowed) and/or with medical conditions that would limit the ability to increase protein intake such as kidney disease were excluded.

### 2.1. Measurement

Malnutrition–sarcopenia syndrome in this study was defined as the concurrent presence of both malnutrition and sarcopenia in older adults.

### 2.2. Malnutrition Assessment

Nutritional status was assessed using the short-form Mini Nutritional Assessment (MNA) questionnaire, the most widely used tool for nutritional screening and assessment of older adults [14,15]. A recent systematic review and meta-analysis demonstrated that the short-form MNA performed well in older adults with the highest sensitivity and specificity compared to 16 other tools [16]. The questionnaire consists of six questions about dietary regime in the last 3 months, weight loss, immobility, recent stress periods, neuropsychological disorders such as depression or dementia, and body mass index. MNA scores of 12–14 indicate normal nutritional status, 8–11 at risk of malnutrition, and 0–7 malnutrition.

### 2.3. Sarcopenia Assessment

The operational definition of sarcopenia by the European Working Group on Sarcopenia in Older People 2 (EWGSOP2) [1] was used to assess the participants for sarcopenia. According to the EWGSOP2, individuals with low muscle strength combined with a low muscle quantity were considered to have sarcopenia. When low muscle strength, low muscle quantity, and low physical performance are all detected, sarcopenia is considered severe [1].

A tetrapolar bioelectrical impedance spectroscopy (BIS) (SFB7 device; ImpediMed Ltd., Brisbane, Australia) device was used to estimate the appendicular skeletal muscle mass (ASMM). The details of BIS have previously been described [7,8,9,10,11,12,13,14,15,16,17]. BIS measurements were taken between the right wrist and ankle while the participant rested in a supine position. We used the cross-validated equation by Sergi and colleagues (2015) to calculate the ASMM as recommended by the EWGSOP2 [1].

ASMM (kg) = −3.964 + (0.227 × RI) + (0.095 × weight) + (1.384 × sex) + (0.064 × Xc)

The body weight was measured in kilograms. For sex, a value of 1 represented men and a value of 2 represented women. Both Xc (reactance) and RI (resistance index) are obtained from the BIS device. The agreement of this ASMM equation and dual X-ray absorptiometry was good (adjusted R^2^ = 0.92, standard error of estimate (SEE) = 1.14 kg) [18]. According to the EWGSOP2, a low muscle mass was defined as less than 20 kg for men and less than 15 kg for women [1].

Handgrip strength was measured using smart a Jamar Smart Digital Hand Dynamometer^®^ (Patterson Medical Inc., Warrenville, IL, USA) [19]. During the test, the participant was in a sitting position, with elbow and forearm resting on the chair arm. The mean score of the three trials was analyzed. According to EWGSOP2, a low handgrip strength was defined as less than 27 kg for men and less than 16 kg for women [1].

Gait speed was assessed as part of the short physical performance battery (SPPB) test [20]. The SPPB test includes measures of a 4 m gait speed, time needed to rise from a chair five times, and three standing balance tests, each held for 10 s, and the stances are progressively more difficult. For gait speed, participants were asked to walk at their usual pace over a 4 m course. Canes or walkers were allowed during the walking test, if necessary. Two trials were completed, and the faster time was analyzed. According to the EWGSOP2, a low gait speed was defined as ≤0.8 m/s [1]. The SPPB has an overall score range of 0 to 12, with 0 indicating the lowest physical performance, and a score of 12 indicating the highest performance.

### 2.4. Sedentary and Physical Activity Behavior Assessment

The Actigraph GT3X+ accelerometer (Actigraph, Inc., Pensacola, FL, USA) was used to objectively measure sedentary and physical activity levels. The accelerometer was enclosed in a belt worn on the waist and used for all waking hours for seven consecutive days. The accelerometer was initialized to collect second-by-second activity counts that were scored as minutes spent across the 7 days in intensity levels of sedentary activities (0 to 100 counts), light physical activity (LPA, 101 to 1951 counts), moderate physical activity (MPA, 1952 to 5924 counts), vigorous physical activity (VPA, >5925 counts) [21,22]. Time spent in moderate- and vigorous-intensity activity (MVPA) was defined as accelerometer counts per minute of 1952 and higher. The data were scored and interpreted using the “Actilife software 6.13.4.” An average of at least 10 h of data for a four-day count was required. At least 60 min of continuous zero counts was defined as a non-wearing period and was subsequently removed from the analysis.

### 2.5. Protein and Caloric Intake Assessment

Dietary protein and caloric intake were assessed using the seven abbreviated Block Brief 2000 Food Frequency Questionnaires (FFQ) [23,24]. The questionnaire consists of 70 food items and captures information about dietary intake during the previous year. Raw data captured in the FFQs were analyzed by NutritionQuest (Berkeley, CA, USA) and translated into quantitative intake of macronutrients and micronutrients including protein and caloric intake. Protein intake adjusted for body weight was calculated and dichotomized into: meets recommendation or does not meet recommendation [25]. Caloric intake adjusted for age and sex was calculated and dichotomized into: meets recommendation or does not meet recommendation [26].

### 2.6. Self-Efficacy for Physical Activity Assessment

Self-efficacy for physical activity was assessed by the Physical Activity Assessment Inventory [27]. This is a 13-item numeric scale that asks respondents to rate how confident they are that they could perform their usual physical activities in different circumstances. Response options range from 0 (cannot do at all) to 100 (certain can do) in increments of 10, with higher scores indicative of increased levels of self-efficacy. Cronbach’s alpha was 0.95, and content validity was supported in previous studies [27,28].

### 2.7. Aging Expectations Assessment

The Expectations Regarding Aging (ERA-38) survey [29] was used to assess aging expectations. The survey was developed to measure the extent to which individuals expect to experience age-associated decline and has been used to examine the relationship between perceptions of aging, health behaviors, and outcomes. It includes 38 questions related to several domains, including general health, physical and mental health, cognitive function, and independence in activities of daily living. Higher average scores indicate more positive expectations and lower scores indicate expected decline in health and functional and mental status. The total scores were used in the analysis. This survey showed acceptable reliability and validity in a previous study in older adults [29].

### 2.8. Other Measures

Demographics survey data on marital status, education level, falls, and smoking status were collected. Data on the physical- and mental-health-related quality of life was measured by the SF-36 [30] and the Timed and Up and Go (TUG) test was administered [31].

### 2.9. Statistical Analysis

Assuming a proportion of 20% based on a previous study [32], a precision of 10%, and an estimated design effect of 2 to account for differences between the CCRCs, a sample size of 122 participants was required. Statistical analyses were performed with SPSS software version 27. Descriptive statistics were used to describe the study variables. We present the categorical data as absolute number and percentages (%) and the continuous data as the mean (M) standard deviation (SD). We applied the Pearson chi-squared and Fisher’s exact test for categorical data and the independent samples t-test for continuous data to compare the differences between participants with and without MSS. Responses to the items on the SF-36 survey were compiled using standard procedures to obtain t-scores for the physical and mental composite scales [30]. Univariate logistic regression analysis was performed for each variable (protein intake, caloric intake, sedentary time, LPA, MVPA, self-efficacy, and ERA) to check for significant association with MSS. A multiple logistic regression was also conducted to examine the relationship between these variables and MSS. Stepwise model selection with entry and removal criteria of *p* < 0.05 and *p* > 0.10, respectively, was used and all logistic regression models were adjusted for age and gender. Statistical significance was defined as *p* < 0.05. Participants with missing data were excluded from analyses (*n* = 9).

## 3. Results

### 3.1. Characteristics of Study Sample

A total of 96 participants were included in the analyses. The mean age of the study participants was 82.5 (SD = 7.4) years (range from 70 to 99 years), and 79 participants (82.3%) were female. The majority of participants were White or Caucasian (*n* = 78; 81.3%), widowed (*n* = 57; 59.4%), highly educated with some college or above (*n* = 68; 70.8%), and non-current smokers (*n* = 85; 88.5%). The fall incidence was high in our sample—43 (44.8%) participants had had at least one fall in the past 12 months. Only 42 (43.8%) participants met the daily recommendation for protein intake and 33 (34.4%) met the daily recommendation for caloric intake. The sample also showed low muscle mass, strength, function, LPA, and MVPA, and high sedentary time. The average physical (23.3; SD = 0.30) and mental (12.6; SD = 0.3) HRQoL scores were substantially lower than the lower 25th percentile for persons aged 75 and above. All characteristics of the participants are depicted in Table 1.

### 3.2. Sarcopenia, Malnutrition, and MMS

Based on the EWGSOP2 criteria, 36 participants (37.5%) were found to have sarcopenia. Based on the results of the MNA scale, the prevalence of malnutrition risk and malnutrition were 21.9% (*n* = 21) and 13.4% (*n* = 13), respectively. When considering both sarcopenia and malnutrition syndromes, 12.5% (*n* = 12) of the participants had MSS (Table 1).

### 3.3. Comparison of Characteristics between with MSS and without MSS

The general characteristics and all other variables were compared between those with and without MSS (Table 1). Compared to participants without MSS, participants with MSS had lower handgrip strength (*p* < 0.001), ASMM (*p* < 0.001), gait speed (*p* = 0.016), SPPB (*p* = 0.003), and TUG (*p* = 0.001). Participants with MSS also showed higher sedentary time (*p* < 0.001) than those without MSS (Table 1). Participants without MSS represented higher levels of LPA, MVPA, self-efficacy, ERA, and physical and mental HRQoL, although without reaching significance (Table 1).

### 3.4. Factors Associated with MMS

The time spent in sedentary behaviors showed a positive association with MSS. Participants who spent more time in sedentary behaviors were more likely to have MSS (odds ratio (OR) = 1.024; 95% confidence interval (CI): 1.010–1.039) compared to those who did not. The binary logistic regression models, including each independent variable and MSS, are presented in Table 2. All regression models were adjusted for age and gender. The multiple logistic regression analysis adjusted for age and gender demonstrated that high time spent in sedentary behaviors (OR = 1.041; 95% CI: 1.011–1.071) was significantly associated with higher odds of having MSS and that CCRC residents with high expectations regarding aging (OR = 0.896; 95% CI: 0.806–0.997) were less likely to have MSS than those with low expectations regarding aging (Table 3).

## 4. Discussion

This study is the first to examine the prevalence of MSS and its relationship to self-management processes including self-efficacy and aging expectations as well as the sedentary, physical activity, and dietary self-management behaviors in older adults living in CCRCs. The findings demonstrated that sarcopenia, malnutrition, and MSS were prevalent in CCRC residents and those with MSS have lower handgrip strength, muscle mass, gait speed, and SPPB score compared to those without MSS. CCRC residents with MSS also demonstrated higher sedentary time and higher score on the TUG test (i.e., indicates poor performance) compared to those without MSS. We also found that high time spent in sedentary was associated with higher odds of having MSS among CCRC residents and those with high expectations regarding aging were less likely to have MSS than those with low expectations regarding aging, after adjusting for age and gender.

To the best of our knowledge, the prevalence of MSS and this study’s variables have not been examined among CCRC residents. However, limited studies examined the prevalence of MSS among older adults in other settings such as hospitals, rehabilitation facilities, and nursing homes [33,34,35]. The prevalence of MSS in our study was higher compared to two recent studies conducted among hospitalized older adults and nursing home residents. Both studies used the EWGSOP2 criteria for sarcopenia (using bioimpedance analysis (BIA) for estimating muscle mass) and MNA criteria for malnutrition and showed MSS prevalence of 4.6% (*n* = 350) [33] and 5% (*n* = 92) [35], respectively. The prevalence in our study was also higher than that in a previous report of an Asian population where the Asia Working Group for Sarcopenia (AWGS) criteria for sarcopenia (using an equation validated in a Chinese population for estimating muscle mass) and MNA criteria for malnutrition were used (*n* = 453; MSS = 5.5%) [34]. However, the prevalence in our study was lower than that in a Japanese study which investigated the geriatric rehabilitation of inpatients (*n* = 601, 23.5%) and employed AWGS criteria (using BIA for estimating muscle mass) and the Global Leadership Initiative on Malnutrition criteria for sarcopenia and malnutrition, respectively [32]. These differences in the prevalence of MSS can be attributed to the varied definitions of sarcopenia and malnutrition, population characteristics, settings, and assessment tools across the studies. These results suggest that it is important to screen for and address sarcopenia and malnutrition and employ up-to-date criteria and consistent assessment tools for both sarcopenia and malnutrition.

In this study, a longer period of sedentary time was associated with MSS. While literature exists on the relationship between physical activity and sarcopenia [36,37,38], to our knowledge, limited literature has examined the association between sedentary behavior and sarcopenia and no other literature has examined the relationship between sedentary behavior and MSS in older adults, particularly CCRC residents. In this context, it is important to emphasize that sedentary behavior is not physical inactivity and is defined as any waking behavior characterized by an energy expenditure ≤1.5 METs while in a sitting, reclining, or lying posture [39]. Importantly, studies showed that older adults spend the majority of their waking day in sedentary activities [40], and that sedentary behavior has been linked to higher levels of adiposity that results in a catabolic effect on muscle by promoting protein degradation [41] and sarcopenia [42]. Hence, it is plausible that sedentary behavior is associated with MSS as demonstrated by our study.

Another associated factor with MSS was aging expectations. Aging expectations is a modifiable factor and defined as the expectations of achievement and maintenance of physical, mental, and cognitive health [29]. Older adults’ expectations of aging have been reported to influence current health behaviors and future health [43,44]. Older adults who expect that symptoms of illness and health problems are part of aging are less likely to seek treatment and care and less willing to engage in self-management and health-promoting behaviors [44,45,46]. This is consistent with our finding on the association between MSS and aging expectations which suggests that expectations of aging may be an important consideration in understanding health behaviors of older adults that could contribute to MSS. While studies showed that high expectations of aging have been found to influence health behaviors, older adults with low aging expectations reported fewer health-maintenance behaviors such as engaging in physical activity, eating a healthy, nutritious diet, and making health a priority [46,47,48]. Furthermore, our findings did not show a relationship between caloric and protein intake and MSS. While there is lack of studies on the association between caloric and protein intake and MSS among older adults including CCRC residents, it is reported that adequate caloric and protein intake is necessary for the formation and maintenance of muscle mass and malnutrition includes an imbalance of energy and protein that could lead to negative effects on body composition and clinical outcomes [49]. One possible explanation for our unexpected finding could be related to the self-reported survey used to assess caloric and protein intake.

Our findings have several important implications. Our study demonstrated significant co-existing of sarcopenia and malnutrition, and that sedentary behaviors and low aging expectations are highly prevalent and associated with MSS among CCRC residents, which can lead to deleterious health outcomes including increased mortality rates [34]. These findings underscore the importance of early screening and detection of both sarcopenia and malnutrition not only among CCRC residents but even among young older adults. Since a decline in muscle mass and strength starts after early adulthood [50], screening after this age period may be an effective strategy to prevent or delay the development of MSS in CCRC residents and those moving to more restrictive living environments such as nursing homes. Moreover, future studies should examine the relationship between caloric and protein intake and MSS among older adults, particularly CCRC residents. Furthermore, early self-management interventions to decrease sedentary behavior, increase physical activity, and promote dietary intake including protein and caloric intake are needed to prevent and/or mitigate MSS among young and very old adults. Interventions could be more effective if the self-management-process factors such as aging expectations were targeted. Lastly, it is valuable for health-care professionals to understand and be aware of how an older individual’s expectations of aging influence different health-promoting behaviors such as physical activity and healthy-eating behavior.

This study has several strengths and limitations that should be considered when interpreting its results. Our study was the first to combine a multidimensional analysis of malnutrition with the analysis of sarcopenia, including using bioelectrical impedance spectroscopy and EWGSOP2 criteria, in an understudied and vulnerable group of CCRC residents. Another strength of our study was the exploration of self-management-process factors and behaviors and their relationship to MSS in CCRC populations. Limitations included that the sample size used in this study was relatively small, which may limit the generalizability of the study and underestimate the prevalence of MSS in this setting. Due to the nature of study design, it is difficult to derive causal relationships between the study variables and MSS from cross-sectional analysis. Another limitation is the BIS method which can be misleading because of the common hydration problems among older adults. However, it is inexpensive, safe, and well-correlated with MRI and DXA predictions. Furthermore, while the FFQ represents an easy, suitable, and cost-effective tool for assessing nutrient intakes in older adults, the questionnaire relies on memory and is prone to measurement errors.

## 5. Conclusions

Our results extend current knowledge by demonstrating that clinical presentation of malnutrition, sarcopenia, and MSS are prevalent in older adults living CRCCs. We suggest that screening for malnutrition and sarcopenia and identifying MSS should be conducted in this high-risk group. We also found that increased time spent in sedentary behaviors was associated with MSS in older adults living CRCCs and those with high aging expectations were less likely to have MSS than those with low expectations regarding aging. Future research should attempt to further understand other risk factors for MSS, and the relationship between MSS and adverse health outcomes such as disability and frailty. Designing and implementing self-management interventions that consider the self-management-process factors is needed to prevent MSS and mitigate its negative health outcomes among older adults living in CRCCs.

## Figures and Tables

**Table 1:** Characteristics of the total sample and comparison between those with and without MSS.

Characteristic	Total Sample (*n* = 96, 100%)	No MSS (*n* = 84, 87.5%)	MSS (*n* = 12, 12.5%)	*p*-Value (No MSS vs. MSS)
Age (years)	82.5 ± 7.4	81.9 ± 7.2	86.1 ± 8.4	0.074
BMI	26.1 ± 6.1	27.12 ± 5.8	20.26 ± 1.2	<0.001
Gender				0.686
Female	79 (82.3)	68 (81.0)	11 (91.7)	
Male	17 (17.7)	16 (19)	1 (8.3)	
Race				0.207
White or Caucasian	78 (81.3)	68 (81.0)	10 (83.3)	
Black or African American	16 (16.7)	15 (17.9)	1 (8.3)	
American Indian/Alaska Native	2 (2.1)	1 (1.2)	1 (8.3)	
Marital Status				0.298
Never married or single	4 (4.2)	3 (3.6)	1 (8.3)	
Married	18 (18.8)	18 (21.4)	0 (0)	
Divorced or separated	17 (17.7)	14 (16.7)	3 (25.0)	
Widowed	57 (59.4)	49 (58.3)	8 (66.7)	
Education Level				0.742
High school or below	28 (29.2)	24 (28.6)	4 (33.3)	
College and above	68 (70.8)	60 (71.4)	8 (66.7)	
Falls				0.313
Yes	43 (44.8)	36 (42.9)	7 (58.3)	
No	53 (55.2)	48 (57.1)	5 (41.7)	
Smoking Status				0.624
Current smokers	11 (11.5)	9 (10.7)	2 (16.7)	
Non-current smokers	85 (88.5)	75 (89.3)	10 (83.3)	
Nutrition Status				<0.001
Normal	62 (64.6)	62 (73.8)	0 (0)	
Malnutrition risk	21 (21.9)	21 (25.0)	0 (0)	
Malnutrition	13 (13.4)	1 (1.2)	12 (100)	
Sarcopenia Status				0.004
No	60 (62.5)	60 (71.4)	0 (0)	
Yes	36 (37.5)	24 (28.6)	12 (100)	
Protein Intake				0.437
Yes	42 (43.8)	38 (45.2)	4 (33.3)	
No	54 (56.3)	46 (54.8)	8 (66.7)	
Caloric Intake				0.167
Yes	33 (34.4)	31 (36.9)	2 (16.7)	
No	63 (65.6)	53 (63.1)	10 (83.3)	
Number of chronic conditions	4.8 ± 5.4	4.6 ± 5.4	5.6 ± 5.3	0.598
Handgrip strength (kg)	19.0 ± 6.9	19.9 ± 6.7	11.9 ± 3.7	<0.001
Body fat %	37.5 ± 7.2	39.1 ± 5.5	37.26 ± 7.4	0.403
ASMM (kg)	16.0 ± 3.6	16.4 ± 3.7	13.2 ± 2.1	<0.001
Gait speed (m/s)	0.7 ± 0.2	0.7 ± 0.2	0.57 ± 0.2	0.016
SPPB	7.9 ± 2.6	8.2 ± 2.5	5.83 ± 2.7	0.003
TUG	14.9 ± 5.4	14.2 ± 4.4	19.8 ± 8.4	0.001
Sedentary time	519.1 ± 77.8	506.7 ± 73.5	608.5 ± 41.5	<0.001
LPA	151.6 ± 54.6	153.6 ± 55.8	137.2 ± 43.9	0.353
MVPA	4.6 ± 10.2	5.1 ± 10.9	2.1 ± 2.0	0.369
Self-efficacy	919.9 ± 256.9	928.7 ± 251.3	858.6 ± 298.1	0.379
ERA	40.1 ± 14.9	40.8 ± 15.3	35.4 ± 10.8	0.146
HRQoL				
Physical	23.3 ± 0.3	23.3 ± 0.3	23.2 ± 0.3	0.170
Mental	12.6 ± 0.3	12.7 ± 0.4	12.4 ± 0.3	0.069

Data are presented as the number (percent) for the following variables: gender, marital status, education level, falls, smoking status, nutritional status, sarcopenia status, meet daily protein intake recommendations, and meet daily caloric intake recommendations. For other variables, the mean ± SD are used. The independent sample t-test was used for the continuous variables, and the Pearson chi-square or Fisher’s exact test was used for categorical variables. During testing, *p* < 0.05 was considered statistically significant. MSS: malnutrition–sarcopenia syndrome; n: sample size; BMI: body mass index; ASMM: appendicular skeletal muscle mass; SPPB: short physical performance battery; TUG: Timed Up and Go; LPA: light physical activity; MVPA: moderate and vigorous physical activity; ERA: expectations regarding aging.

**Table 2:** Binary logistic regression models including each independent variable and MSS.

	Coefficient	SE	Wald	*p*-Value	OR	95% CI
Protein intake	−0.140	0.685	0.42	0.838	0.869	0.227–3.327
Caloric intake	−0.980	0.825	1.411	0.235	0.375	0.074–1.891
Sedentary time	0.024	0.007	10.878	0.001	1.024	1.010–1.039
LPA	−0.003	0.007	0.269	0.604	0.997	0.984–1.009
MVPA	−0.089	0.111	0.651	0.420	0.915	0.736–1.136
Self-efficacy	−0.002	0.001	2.012	0.156	0.998	0.995–1.001
ERA	−0.030	0.024	1.560	0.212	0.970	0.925–1.017

MSS: malnutrition–sarcopenia syndrome; SE: standard error; OR: odds ratio; CI: confidence interval; LPA: light physical activity; MVPA: moderate and vigorous physical activity; ERA: expectations regarding aging.

**Table 3:** Factors influencing MSS by multiple logistic regression analysis.

	Coefficient	SE	Wald	*p*-Value	OR	95% CI
Protein intake	−2.115	1.513	1.954	0.162	0.121	0.006–2.341
Caloric intake	−1.642	1.421	1.335	0.248	0.194	0.012–3.137
Sedentary time	0.040	0.015	7.409	0.006	1.041	1.011–1.071
LPA	−0.021	0.013	2.697	0.101	0.980	0.956–1.004
MVPA	−0.015	0.126	0.013	0.908	0.986	0.770–1.262
Self-efficacy	−0.006	0.004	2.173	0.140	1.006	0.998–1.013
ERA	−0.110	0.054	4.085	0.043	0.896	0.806–0.997

MSS: malnutrition–sarcopenia syndrome; SE: standard error; OR: odds ratio; CI: confidence interval; LPA: light physical activity; MVPA: moderate and vigorous physical activity; ERA: expectations regarding aging.

## Data Availability

Data can be requested from the corresponding author (with justification).
